# Reduced uptake of [11C]‐ABP688, a PET tracer for metabolic glutamate receptor 5 in hippocampus and amygdala in Alzheimer’s dementia

**DOI:** 10.1002/brb3.1632

**Published:** 2020-04-18

**Authors:** Valerie Treyer, Anton F. Gietl, Husam Suliman, Esmeralda Gruber, Rafael Meyer, Andreas Buchmann, Anass Johayem, Paul G. Unschuld, Roger M. Nitsch, Alfred Buck, Simon M. Ametamey, Christoph Hock

**Affiliations:** ^1^ Institute for Regenerative Medicine (IREM) University of Zurich Schlieren Switzerland; ^2^ Department of Nuclear Medicine University Hospital of Zurich Zurich Switzerland; ^3^ Hospital for Psychogeriatric Medicine University of Zurich Zurich Switzerland; ^4^ Neurimmune Schlieren Switzerland; ^5^ Institute of Radiopharmaceutical Sciences ETH Zurich Zurich Switzerland

**Keywords:** ABP688, Alzheimer's dementia, amygdala, hippocampus, metabolic glutamate receptor, mGLUR5

## Abstract

**Introduction:**

Metabotropic glutamate receptors play a critical role in the pathogenesis of Alzheimer's disease due to their involvement in processes of memory formation, neuroplasticity, and synaptotoxity. The objective of the current study was to study mGluR5 availability measured by [^11^C]‐ABP688 (ABP) in patients with clinically diagnosed Alzheimer's dementia (AD).

**Methods:**

A bolus‐infusion protocol of [^11^C]‐ABP688 was applied in 9 subjects with AD and 10 cognitively healthy controls (Controls) to derive distribution volume estimates of mGluR5. Furthermore, we also estimated cerebral perfusion by averaging early frame signal of initial ABP bolus injection.

**Results:**

Subjects with Alzheimer's dementia (mean age: 77.3/*SD* 5.7) were older than controls (mean age: 68.5/*SD*: 9.6) and scored lower on the MMSE (22.1/SD2.7 vs. 29.0/SD0.8). There were no overall differences in ABP signal. However, distribution volume ratio (DVR) for ABP was reduced in the bilateral hippocampus (AD: 1.34/*SD*: 0.40 vs. Control: 1.84/*SD*:0.31, *p* = .007) and the bilateral amygdala (AD:1.86/*SD*:0.26 vs. Control:2.33/*SD*:0.37 *p* = .006) in AD patients compared to controls.

Estimate of cerebral blood flow was reduced in the bilateral hippocampus in AD (AD:0.75/*SD*:0.10 vs. Control:0.86/*SD*:0.09 *p* = .02).

**Conclusion:**

Our findings demonstrate reduced mGluR5 binding in the hippocampus and amygdala in Alzheimer's dementia. Whether this is due to synaptic loss and/or consecutive reduction of potential binding sites or reflects disease inherent mechanisms remains to be elucidated in future studies.

## Graphical abstract

The objective of the current study was to study mGluR5 availability measured by [11C]‐ABP688 in patients with clinically diagnosed Alzheimer’s dementia. Our findings demonstrate reduced mGluR5 binding in the hippocampus and amygdala in Alzheimer’s dementia.

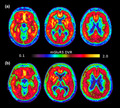

## 1 INTRODUCTION

The pathology of Alzheimer's disease can be divided in accumulation of aggregated proteins, particularly extracellular beta‐amyloid and intracellular phosphorylated tau, losses of synapses and neurons and reactive processes including activation of microglia, astrocytosis and regenerative processes of undetermined significance (Duyckaerts, Delatour, & Potier, 2009). Beta‐amyloid plaques and tau pathology are seen as hallmarks of the disease and are applied for pathological staging (Boluda et al., 2014; Braak & Braak, 1995). In addition, synaptic dysfunction and synaptic loss reportedly correlate strongly with cognitive decline (Scheff, Price, Schmitt, & Mufson, 2006; Terry et al., 1991)*.* Both Abeta‐ and tau pathology contribute to synaptic dysfunction in Alzheimer's disease and exert a detrimental interaction at the synaptic level as reviewed by Forner, Baglietto‐Vargas, Martini, Trujillo‐Estrada, & LaFerla, 2017 (Forner et al., 2017).

One of the puzzle stones being involved in synaptic toxicity in AD is the interaction of Abeta‐pathology with metabotropic glutamate receptor 5 (mGluR5). mGluR5 together with mGluR1 belongs to the subgroup I of metabotropic glutamate receptors which are all G‐protein coupled receptors. Subgroup I is functionally linked to polyphosphoinositide hydrolysis and negatively coupled with K+ channels (Caraci, Nicoletti, & Copani, 2018). It was shown that group I mGluRs were involved in Abeta induced synaptic long‐term depression (Chen et al., 2013).

With respect to synaptotoxicity, mGluR5 was necessary for cluster formation of Abeta‐oligomers (Abetao) at the synaptic plasma membrane which in turn leads to altered redistribution of mGluR5 and aberrant Ca2+ mobilization (Renner et al., 2010). It mediated increases in intracellular Ca2+ and dendritic spine loss via complexes of prion protein and Abetao (Um et al., 2013). Furthermore, Abeta42 was shown to overactivate mGluR5 with a consequence of increased Ca2+ storage in the endoplasmic reticulum and mushroom spine loss in hippocampal neurons (Zhang et al., 2015).

mGluR5 is located mainly postsynaptically in the nerve cells throughout the CNS where they locate close to ionotropic glutamate receptors, but are also expressed in astrocytes and microglial cells (Caraci et al., 2018).

The mGluR5 receptor can be assessed in vivo via the PET Tracer 3‐(6‐methylpyridin‐2‐ylethynyl)‐cyclohex‐2‐enone‐O‐11 C‐methyl‐oxime ([11C]‐ABP688 or ABP from now on)(Ametamey et al., 2007). Ex vivo and in vivo studies using rodents showed specific binding in mGluR5‐rich brain regions, which was also corroborated by studies in mGluR5 knockout mice (Ametamey et al., 2006). Findings in humans included reduced mGluR5 binding in younger subjects with depression (Deschwanden et al., 2011) while others could not identify such a reduction in late‐life depression (DeLorenzo et al., 2015). One study found a strong mGluR5 reduction in smokers and ex‐smokers compared to nonsmokers and a positive age correlation in putamen and occipital lobe over all groups (Akkus et al., 2013). In the individual subgroups of smokers or ex‐smokers, positive correlations were also seen in other regions including amygdala. When taking into account number or years of smoking in the smoker group, only putamen remained significant with age as significant covariate. No correlation was seen in the nonsmoker group.

In neurodegenerative disease, widespread reductions in ABP signal were identified in frontotemporal dementia (Leuzy et al., 2016).

mGluR5‐PET was used in two mouse models of Alzheimer's disease. One study using ABP failed to find changes in mGluR5 binding in APP transgenic mice (tg‐ArcSwe) compared to wild‐type mice (Fang et al., 2017). Another study discovered reduced mGluR5 assessed by 18F‐2‐fluoro‐6‐(3‐fluorophenylethynyl)‐pyridine (FPEP) (18‐F‐FPEP) binding in a more aggressive transgenic mouse model (M. Lee et al., 2018).

Here, we report an exploratory study of mGluR5 in Alzheimer's dementia (AD) in humans. We examined nine subjects with AD and ten cognitively healthy controls with a bolus‐infusion protocol of ABP. In addition, we have assessed the early frame signal of ABP as an estimate of cerebral blood flow (Treyer et al., 2007). Due to the involvement of mGluR5 in Alzheimer's pathogenesis, we expected to see changes in mGluR5 binding especially in mediotemporal structures. Furthermore, we expected reduced perfusion‐related signal of ABP in subjects with Alzheimer's disease, as perfusion correlates with neuronal activity and cognitive performance, and is known to be reduced in Alzheimer's disease (Gietl et al., 2015; Rostomian, Madison, Rabinovici, & Jagust, 2011).

## 2 MATERIALS AND METHODS

### 2.1 Study population

Nine patients with probable Alzheimer dementia (AD group) and 10 healthy controls (HCS group) were enrolled into the study. Enrolment took place between 2010 and 2012, and final data analysis was protracted due to changes in study personnel. Probable Alzheimer's dementia was diagnosed clinically according to NINCDS‐ADRDA criteria (McKhann et al., 1984) and ICD‐10 (Dilling, 2006). Main inclusion for the AD group was a Mini‐Mental State Examination (MMSE) (Folstein, Folstein, & McHugh, 1975) between 18 and 26 and a Clinical Dementia Rating (CDR) (Hughes, Berg, Danziger, Coben, & Martin, 1982) (Morris, 1993) global score below 2 (inclusive). To support clinically diagnosed AD perfusion and voxel‐based morphometry maps, analysis was performed with SPM12 (Dartel Toolbox) to confirm a typical AD biomarker pattern in the AD group. Healthy subjects were only included if they scored higher than 28 points on the MMSE (inclusive). All subjects ought to have capacity to consent to study procedures.

Participants were examined by a study physician to ensure good health condition. Subjects with drug or alcohol abuse and acute psychiatric disease were excluded. The Montgomery Asberg Depression Scale (MADRS) (Montgomery & Asberg, 1979) was applied to check for symptoms of depression. In the AD group, six subjects were on cholinesterase inhibitors, 4 subjects received escitalopram and one mirtazapine. Further exclusion criteria were contraindications for magnet resonance tomography and significant exposure to radiation. Two subjects had increased thyroid‐stimulating hormone values but did not show altered peripheral thyroid hormones.

Neuropsychological assessment included CERAD PLUS (Thalmann et al., 1997), fluency tests (Regard, Strauss, & Knapp, 1982) (S‐words and 5‐point test), the German version of Auditory Verbal Learning test called VLMT (Helmstädter & Lux, 2001), visual pair learning test, digit span, corsi block forward and backward as included in the German version of the Wechsler Memory Scale revised version (Haerting et al., 2000), trail making test TMT A and B (Reitan, 1955), Stroop's test (Stroop, 1935), and a test to find concepts for similarities between different symbols on cards (Kramer)(Kramer, 1954).

The study was conducted after approval of the ethics committee of the canton Zurich in accordance with the local law and the Declaration of Helsinki (WorldMedicalAssociation, 2008).

### 2.2 Manual volumetry of the hippocampus

To assess directly hippocampus volume, manual outlining by a trained volumetrist was performed on MRI images to ensure appropriate depiction of the dementia‐related atrophy. For correction of whole‐brain volume, the hammers atlas derived gray and white matter volumes as well as ventricles were summed.

### 2.3 PET analysis

All participants received a 3D T1 anatomical scan (resolution 0.5 × 0.5 × 1 mm) on a Philips Achieva 3T Scanner. PET scans were performed using standard bolus‐infusion protocol (Burger et al., 2010). Images were acquired dynamically over 60 min and reconstructed using standard filtered‐back‐projection algorithm on whole body PET/CT scanners Discovery STE or RX PET/CT (GE Healthcare). Due to the bolus‐infusion paradigm used for injection, an equilibrium was reached after 40 min. As shown in previous publications (Burger et al., 2010; Treyer et al., 2007), images acquired under equilibrium condition can be used to directly quantify DVR values by dividing with the cerebellum values. Participants received an average injection dose of 551.9 MBq (±59.3) [11C]‐ABP688. Details about tracer production can be found in the original publication (Ametamey et al., 2007).

Images were analyzed with PMOD 3.6 Neuro Tool and deep nuclear parcellation method as implemented in Neurotool. MRI and PET images were coregistered based on early frames signal (first 6 min). Gray–white matter segmentation on the MRI images was performed with standard 50% probability. Only gray matter segmented regions in the coregistered PET image were analyzed to reduce potential biases of atrophy. Cortical region of interests were defined using Hammers Maximum Probability Atlas while noncortical regions were calculated with the deep nuclei parcellation method. For this study, a knowledge base of 20 reference sets was selected. Regions of interest as calculated by the methods mentioned above were applied on PET and MRI images in individual MRI spaces and were controlled individually and adapted if necessary by an expert in imaging processing. Individual cortical regions as defined by the atlas were averaged between hemispheres and lobes to test for general differences in the lobes. White matter and ventricular regions were calculated in order to appropriately apply volume of interest (VOI)‐based partial volume correction (geometric transfer matrix method) on the individual regions of interests as implemented in PMOD Neuro Tool and taking care of spillover among all volumes of interest and convolve with point spread function (Rousset, Ma, & Evans, 1998). This correction does take care of reduced signal due to atrophic brain regions. Together both methods, on the one hand the exact outline of the regions of interest on the gray matter image and secondly the volume of interest‐based partial volume correction ensure better quantification and correction of partial volume effects especially due to atrophy for all regions.

For distribution volume estimates (DVR), late frame PET signal (45–60 min) was averaged over the VOIs and divided by cerebellar gray matter values. Perfusion‐related signal was extracted from early frame data (first 3 min) and divided by cerebellar signal for normalization to control for variations in injected dose (Burger et al., 2010). As mentioned above, not all regions available in the templates were used for analysis. Only bilaterally averaged region assessments were used to test for group differences in the cortical regions as no hypothesis on differences within lobes was set. Therefore, the VOIs in frontal, temporal, parietal, and occipital lobe were merged within the lobes (resulting in 4 regions). The remaining regions were taken as defined in the hammers atlas and the parcellation method mentioned above and only averaged between sides. This results in following bilateral regions: insula, anterior cingulate, posterior cingulate, thalamus, putamen, caudate, amygdala, and hippocampus (additional 8 regions). A total of 12 regions was taken into statistical analysis.

Due to the differences in hippocampal volume additionally to the volume of interest‐based approach for partial volume correction, a different MRI‐based approach was selected for methodological reasons in addition. We used the Muller‐Gartner method according PMOD standard implementation for gray matter spill‐out and white matter spill‐in on the dynamic PET images (Muller‐Gartner et al., 1992). We used the results of these methods primarily to test for robustness of the results with the standard VOI‐based method mentioned above (Greve et al., 2016).

### 2.4 Statistical analysis

This is an exploratory study with a sample size of 19 subjects; thus, we chose not to correct for multiple comparisons. All variable of interests were tested for normal distribution using Kolmogorov–Smirnoff test and either parametric or nonparametric tests for comparison of two independent groups were conducted with SPSS 25. VOIs DVR values showed normal distribution. For testing effects of covariates, general linear model univariate procedures were used. Tests were performed two sided. To check for potential biases, also nonpartial volume corrected regional values were statistically tested for significance and showed similar effects.

## 3 RESULTS

Please note that all *p* values are reported uncorrected.

### 3.1 Confirmation of perfusion and volumetric differences between AD and control group

The summed early frames were used as perfusion brain maps positively confirmed AD‐typical reductions in parietal lobe and posterior cingulate in the AD group compared to controls (Herholz, 2011). A direct group comparison performed with SPM 12 (*t* test, uncorrected for multiple testing only due to small sample size) revealing reduction specific in these regions (i.e., posterior cingulate, *T* = 3.73, *Z* = 3.14, *p* = .001, highest voxel in left inferior temporal area (Brodmann Area 20) *T* = 6.13, Z = 4.40, *p* = .000).

**FIGURE 1:**
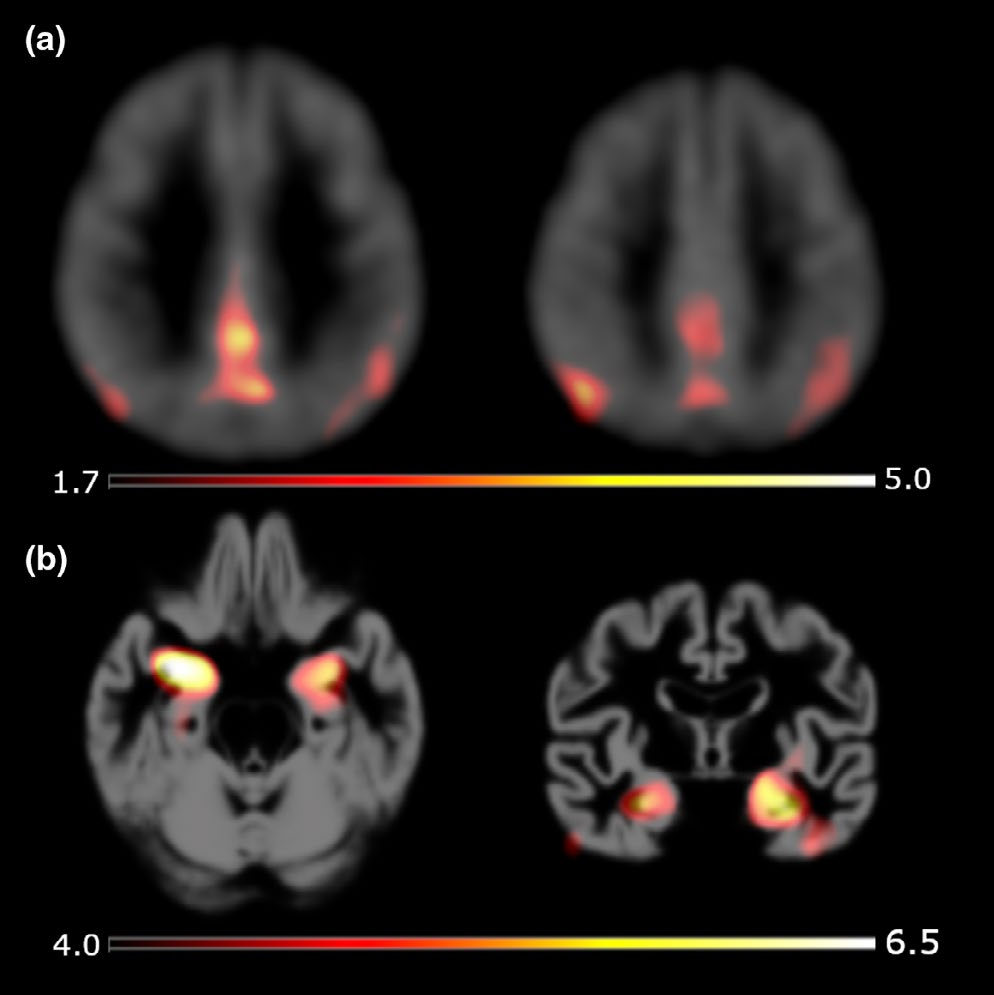
Voxel‐wise group comparisons with perfusion estimate (a) and results of volume‐based morphometry (b). Both statistical T‐maps were overlayed over an average healthy perfusion map (a) and from VBM process derived template (b). For display purpose, the axial image of the VBM assessment was rotated, and therefore, left and right side are switched. Cutoff values *p* = .05 (uncorrected)

To assess volumetric differences between groups, a voxel‐based morphometry (VBM) with the 3D T1 MRI was performed. The main difference is seen in the hippocampus, and a typical region is affected in AD (Dubois et al., 2007). Right and left medial temporal regions have higher signal in control group compared to AD group (right side *T* = 6.68, *T* = 4.62, *p* = .000, left side *T* = 5.57, *Z* = 4.15, *p* = .000). Figure 1 shows both difference maps overlayed on an average PET, respectively, through VBM processing generated template image.

Comparing both groups with regard to manual hippocampal volumetry, we see significant differences (corrected for total brain volume (*t*_(17)_ = −4.2, *p* = .001 for right and *t*_(17)_ = −5.4, *p* = .001 for left side). The total brain volume as used for correcting hippocampal volumetry did not differ between both groups (*t*_(17)_ = −0.08, *p* = .9).

### 3.2 Descriptive analyses

**FIGURE 2:**
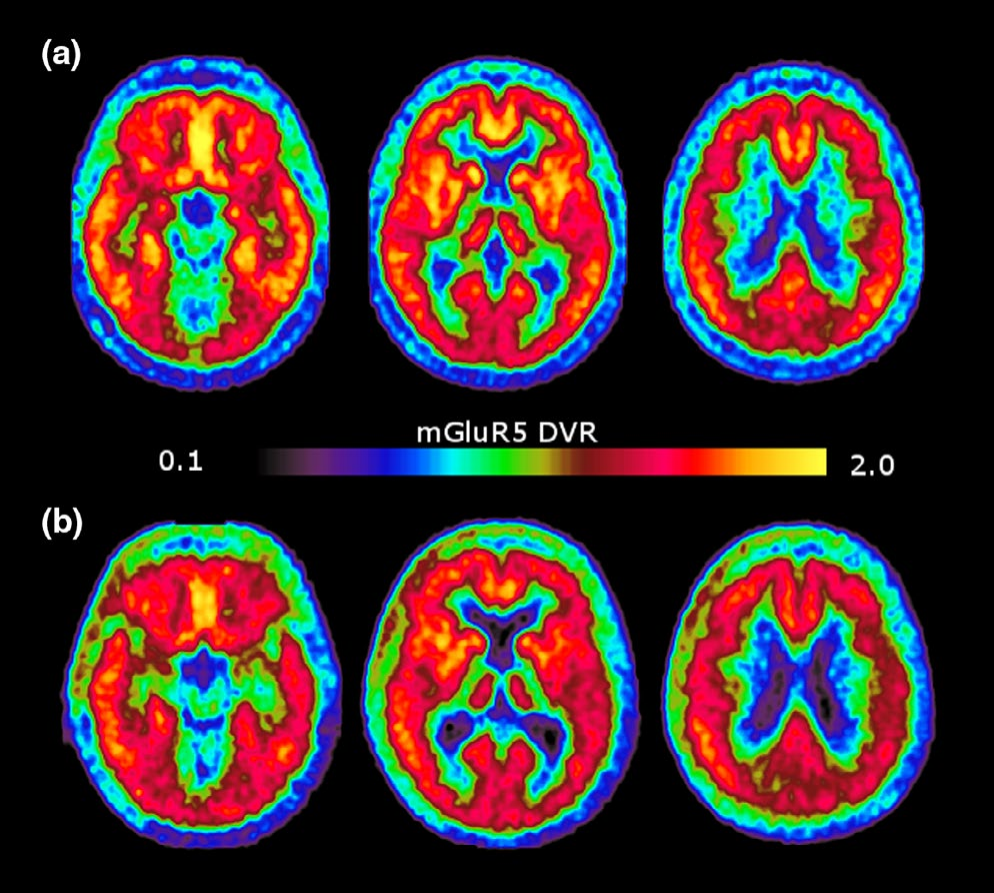
Normalized and averaged mGluR5 DVR images of both groups. (a) shows the axial images of the healthy control group and (b) the images of the AD group

Table 1 summarizes demographics and neuropsychology data with regard to group effects. The results of two neuropsychology tests, TMT, and VLMT are not displayed as only 5 or less subjects in the Alzheimer's disease group could perform or finish these tasks. Figure 2 shows the normalized and within‐group averaged mGluR5 DVR images for healthy controls (A) and AD Group (B).

**TABLE 1:** Description of the study population and comparisons between groups

Parameter	*p* value test for normal distribution (K‐S)	Healthy Controls (*n* = 10)	Alzheimer's disease (*n* = 9)	*p* value *t* test	*p* value Mann–Whitney *U*
Age	.07	68.5 (*SD* 9.6)	77.3 (*SD* 5.7)	.028^*^	
MMSE	.005^*^	29 (*SD* 0.8)	22.1 (*SD* 2.7)		.000^*^
Education in years	.114	13.7 (*SD* 2.8)	11.7 (*SD* 1.8) *N* = 7	.120	
Gender (f/m)		7/3	6/3		
MADRS	.016^*^	2.8 (4.0)	6.2 (2.2)		.028^*^
Boston Naming total	<.001^*^	14.8(*SD* 0.4) (median)	12.1 (*SD* 2.5) (median)		.008^*^
S‐word total	.200	28.7 (*SD* 8.7)	17.6 (*SD* 8.2)	.011^*^	
5‐point total	.200	25.9 (*SD* 8.0)	17.0 (*SD* 5.5)	.012^*^	
WRD recall	.002^*^	8.9 (*SD* 1.0)	1.6 (*SD* 2.6)		.000^*^
WRD recognition	.000^*^	9.9 (*SD* 0.3)	7.6 (*SD* 2.5)		.022^*^
Digit Span forward	.076	7.1 (*SD* 1.4)	5.8 (*SD* 1.9)		.053
Digit Span backward	.016^*^	5.8 (*SD* 1.6)	4.3 (*SD* 1.7)		.053
Corsi Block forward	.011^*^	7.8 (1.3)	6.1 (*SD* 2.4)		.113
Corsi Block backward	.002^*^	6.8 (*SD* 8.3)	5.3 (*SD* 2.0)		.136
Kramer	.200	3.7 (*SD* 1.1)	2.2 (*SD* 1.2)	.011^*^	
WMS visual pairs learning	.200	12.8 (*SD* 2.7)	4.9 (*SD* 3.7) 1 did not perform test	.000^*^	
WMS visual pairs recall	.063	5.2 (*SD* 1.0)	1.4 (*SD* 1.5) 1 did not perform test	.000^*^	
Stroop strong interference/no interference	.038^*^	2.4 (*SD* 0.7) (median 2.2)	2.9 (*SD* 1.2) (median 2.7)		.400
Stroop strong interference	.004^*^	30.2 (*SD* 9.3) (median 27.3)	51.7 (*SD* 26.9) (median 47.0)		.053

^*^ indicates significant values *p*< .05.

As expected, patients with AD showed worse cognitive performance. Six had a CDR global score of 0.5, two of 1 and one scored 2, which was based on the study physician's incorporation of all clinical information in the judgment. All controls had a CDR global score of 0.

The following analyses were performed using the PET‐derived estimates of mGluR5 binding with the VOI‐based partial volume correction as described in the methods section (GMT PVC).

### 3.3 Regional differences in mGluR5 binding in bilateral Hippocampus and Amygdala

A potential overall effect of differences between both groups with regard to estimated mGluR5 distribution was performed including all 12 regions. Overall, there was no between‐group effect with age and depression score as covariates (region as repeated measure factor, *F*_(1.15)_ = 0.013, *p* = .91, partial eta squared (PES) = 0.001). MADRS depression score covariate was significant in this model (*F*_(1,15)_ = 5.6, *p* = .032, PES = 0.273), while covariate age was not significant (*F*_(1,15)_ = 0.277, *p* = .606, PES = 0.018). More details are given in the supplement.

**FIGURE 3:**
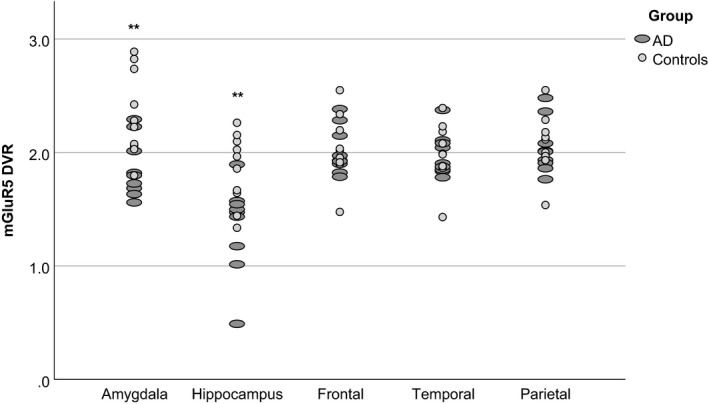
Distribution of DVR values in 5 regions. Bilateral amygdala and hippocampus display reduced mGluR5 binding. Frontal (Frontal), temporal (Temporal), and parietal lobe (Parietal) are displayed as example regions that do not display different binding in patients and healthy controls (see also Table 2)

Group differences in mGluR5 DVR were found in the hippocampus (*t*_(17)_ = −3.04, *p* = .007) and in the amygdala (*t*_(17)_ = −3.16, *p* = .006). The other 10 regions showed no differences (*p* > .4). Further evaluation was only performed for the hippocampus and the amygdala (see also Figure 3 and Table 2). The differences between the two groups were over 20% in the hippocampus and amygdala while in the other regions, especially cortical regions they were clearly below 5% (see Table 2).

**TABLE 2:** mGluR5 binding in the different regions and comparison between groups under study.

Region	HCS average (*SD*) mGluR5 DVR	AD average (*SD*) mGluR5 DVR	delta	delta %	*t*‐value (*df* 17)	*p* value	95% CI
Frontal lobe	2.03 (0.29)	2.02 (0.21)	−0.016	−0.8	−0.14	.890	−0.26–0.23
Temporal lobe	1.99 (0.26)	1.98 (0.19)	−0.008	−0.4	−0.08	.940	−0.23–0.21
Parietal lobe	2.06 (0.27)	2.04 (0.23)	−0.018	−0.9	−0.16^a^	.875	−0.26–0.22
Occipital lobe	1.9 (0.21)	1.97 (0.2)	0.07	3.7	0.74^a^	.469	−0.13–0.27
Insula	1.84 (0.24)	1.93 (0.22)	0.091	4.9	0.85^a^	.406	−0.13–0.32
Anterior Cingulum	2 (0.28)	1.96 (0.26)	−0.044	−2.2	−0.35^a^	.732	−0.31–0.22
Posterior Cingulum	1.77 (0.22)	1.78 (0.17)	0.017	1.0	0.19	.851	−0.17–0.20
Thalamus	1.37 (0.13)	1.37 (0.1)	0.004	0.3	0.07	.946	−0.11–0.12
Putamen	1.81 (0.25)	1.89 (0.21)	0.078	4.3	0.72^a^	.479	−0.15–0.30
Caudate	1.87 (0.4)	1.74 (0.46)	−0.131	−7.0	−0.67	.514	−0.55–0.28
Amygdala	2.33 (0.37)	1.86 (0.26)	−0.472	−20.3	−3.16	**.006**	−0.79 to −0.16
Hippocampus	1.84 (0.31)	1.34 (0.4)	−0.501	−27.2	−3.04	**.007**	−0.85 to −0.15

^a^ Levene's test significant, variances considered as nonhomogenous.

^b^ Bold values are statiscally significants.

To assess for the validity of differences in the hippocampus, additional statistics were performed on uncorrected values (noPVC) and on MRI‐based partial volume corrected images (MG PVC) demonstrating in both cases (noPVC and MG PVC) the effect in the hippocampus (*t*_(17)_ = −2.8, *p* = .012 on uncorrected images and *t*_(17)_ = −2.6, *p* = .017 on MG PVC), revealing the robustness of the results.

One patient in the AD group showed a very low signal in the hippocampus region (lower than cerebellum). The regions were defined on the MRI and not the PET image, and in this case, due to low signal in the PET with some tissue remaining in MRI, a very low signal resulted in this region. This patient had the lowest relative volume of anterior hippocampus. The difference between groups remained significant also when excluding this case in the *t* test analysis of hippocampal values (*t*_(17)_ = −2.85, *p* = .012 instead of *t*_(17)_ = −3.04, *p* = .007 in PVC corrected *t* test).

For further evaluation of hippocampal and amygdala group differences, an univariate analysis of variance with the covariate age and depression (MADRS score) was performed in addition. The model was significant (Hippocampus: *F*_(3,18)_ = 3.9, *p* = .031 partial eta squared (PES) = 0.44, Amygdala: *F*_(3,18)_= 4.7, *p* = .016, PES = 0.49.) The factor group as well as the two covariates were not significant. In the hippocampus, we see following results for Group: *F*_(3,18)_ = 2.25, *p* = .155, PES = 0.13, for age: *F*_(3,18)_ = 0.86, *p* = .368, PES = 0.05 and for MADRS: *F*_(3,18)_ = 1.28, *p* = .277, PES = 0.08.

In the Amygdala, the following results were found for Group: *F*_(3,18)_ = 2.48, *p* = .136, PES = 0.14, for age: *F*_(3,18)_ = 0.35, *p* = .564, PES = 0.02 and for MADRS: *F*_(3,18)_ = 2.92, *p* = .108, PES = 0.16.

### 3.4 Reduced estimate for cerebral perfusion in bilateral hippocampus

Estimate for cerebral perfusion was reduced in the hippocampus (*t*_(17)_ = −2.56, *p* = .020) but not in the amygdala (*t*_(17)_ = −1.28, *p* = .22).

Including age and MADRS as covariate in the univariate model, no significant effects are remaining in hippocampus: *F*_(3,18)_ = 2.8, *p* = .076 Partial Eta Squared = 0.36, Group: *F*_(3,18)_ = 3.3, *p* = .09, PES = 0.18; age *F*_(3,18)_ = 1.0, *p* = .329, PES = 0.06; MADRS: *F*_(3,18)_ = 0.11, *p* = .74, PES = 0.008 and Amygdala: *F*_(3,18)_ = 1.7, *p* = .218, PES = 0.25, Group: *F*_(3,18)_ = 4.8, *p* = .043, PES = 0.25; age *F*_(3,18)_ = 0.49, *p* = .494, PES = 0.03; MADRS: *F*_(3,18)_ = 1.28, *p* = .276, PES = 0.078.

### 3.5 Association of perfusion estimates and mGluR5 binding with cognitive measures

Correlations with neuropsychological variables were conducted only in AD patients for the hippocampus, the region a priori related to cognitive function(Guzman‐Velez, Warren, Feinstein, Bruss, & Tranel, 2016).

Global cognition measured with MMSE score was associated with perfusion estimate (Spearman *ρ* = 0.809, *p* = .008) but not with mGluR5 binding as assessed by DVR (Spearman *ρ* = 0.655, *p* = .055).

We observed significant correlations between verbal fluency and naming (Boston naming and S‐word generation) with the perfusion related (Spearman *ρ* = 0.78, *p* = .013 and r = 0.7, *p* = .036) and the mGluR5 signal (Spearman *ρ* = 0.68, p = .04 and *ρ* = 0.83, *p* = .005).

Kramer test for concept finding (a measure of executive function) also showed a positive correlation with both PET‐derived measures (Spearman perfusion *ρ* = 0.84, *p* = .005, mGluR5 DVR *ρ* = 0.86, *p* = .003).

With respect to memory tests, CERAD word recall performance did correlate with hippocampal mGluR5 DVR (Spearman *ρ* = 0.83, *p* = .006) and perfusion (*ρ* = 0.73, *p* = .027).

## 4 DISCUSSION

We report results on mGluR5 ([11C]‐688ABP) PET in a population of subjects with Alzheimer's disease. To our knowledge, this is the first publication on mGluR5 imaging in human with ABP688 in Alzheimer's disease. We identified lower mGluR5 binding in the bilateral hippocampus and bilateral amygdala. The findings were similar with and without partial volume correction. Due to the small sample size and exploratory character of the study, we focus on effect sizes and not on significance. Controlling for both covariates age and depression revealed higher effect sizes for group effects compared to the covariates in the hippocampus. Furthermore, this region also showed correlations with cognitive measures. In the amygdala, we found an equally high effect for group and the depression score underscoring the relevance of this region also for emotional aspects (Guo et al., 2018; Guzman‐Velez et al., 2016; Leal, Noche, Murray, & Yassa, 2017).

Furthermore, these effects seemed specific to these regions as other regions explored in the study showed no differences in mGluR5 binding and overall binding was not different between groups.

In the hippocampus, we also observed a reduced estimate for cerebral blood flow assessed by the early ABP signal. Furthermore, using a voxel‐wise approach, we found lower perfusion signal in AD‐typical regions, for example, the posterior cingulate.

### 4.1 Reduction in mGluR5 binding may be due to loss in postsynaptic binding sites or hindered ABP binding

A likely explanation for our findings is that loss of neurons and synapses contributes to less postsynaptic mGluR5 binding sites in the hippocampus and the amygdala.

Indeed, we also observe a reduced estimate for cerebral blood flow in the hippocampus. Cerebral blood flow is coupled to cerebral metabolism (Paulson, Hasselbalch, Rostrup, Knudsen, & Pelligrino, 2010) and could serve as an estimate for synaptic integrity (Rocher, Chapon, Blaizot, Baron, & Chavoix, 2003). Our findings are in line with findings in 10‐month‐old male 5xFAD transgenic mice using [^18^F]FPEB to assess mGluR5. Here, a reduction in striatal and hippocampal mGluR5 binding potential was identified together with a reduction of mGluR5 protein levels in the transgenics as compared to age‐matched wild‐type mice. The authors suspected this to be due to excitotoxicity‐mediated synapse loss as this model develops loss of neurons and a reduction of several synaptic markers (Lee et al., 2018). In a longitudinal study, the authors found that in 5xFAD mice showed high fluctuation over time span from 3 to 9 months (Lee et al., 2019). Compared to wild‐type mice, they show significant reduction at 5 months, increased again at 7 months, and decreased again at 9 months of age. The findings suggested that the mGluR5 alterations are best explained due to amyloid‐related neuropathogenesis, which was assessed in parallel.

Findings in frontotemporal dementia, where a reduction in mGluR5 binding overlaps with reductions in FDG‐PET in frontotemporal as well as subcortical regions, support this view further (Leuzy et al., 2016). Also, our results of higher mGLUR5 binding being associated with a better cognitive performance in several tests point in this direction. In addition, another transgenic mouse model overexpressing human APP(ArcSwe) did not exhibit changes in ABP binding potential despite even higher mGluR5 levels measured via immunoblotting and immunofluorescence in the transgenic animals (Fang et al., 2017). In this model, extracellular amyloid deposition starts at around 6 months of age and astrogliosis and tau phosphorylation was described also, but without a profound neuronal or synaptic loss (Lord et al., 2006). Contrary to our findings with ABP in vivo, we have observed increased binding to mGluR5 using PSS232 for autoradiography (Muller Herde, Schibli, Weber, & Ametamey, 2018). At this time point, we cannot fully explain these discrepancies, but suspect the differing experimental conditions (different substance, postmortem via in vivo) to play a role. In this context, Fang et al. (Fang et al., 2017) have already suggested that ABP binding might be hindered by clustering (Renner et al., 2010) and formation of complexes (Um et al., 2013) which could be different for PSS232 or in postmortem tissue.

### 4.2 Reduction in mGluR5 binding as a potential regulatory mechanism

On the other hand, it is striking that we observe reduced mGluR5 binding specifically in the hippocampus and the amygdala regions typically involved early in Alzheimer's disease (Zanchi, Giannakopoulos, Borgwardt, Rodriguez, & Haller, 2017)—but not in other brain regions where we would expect synaptic loss in AD patients (Scheff, Neltner, & Nelson, 2014).

These regions that are highly connected and play an important role in memory formation and in the regulation of emotions (Janak & Tye, 2015; Kitamura et al., 2017; Leal et al., 2017). Thus, it is tempting to speculate whether our findings represent inherent adaptive mechanisms in Alzheimer's disease.

mGluR5 plays important roles in long‐term depression especially in the regions we observe (Bhattacharyya, 2016). This process seems functional for memory formation under physiological conditions. It has been shown that metabotropic glutamate receptor‐dependent long‐term depression was critical for successful aging in rats (H. K. Lee, Min, Gallagher, & Kirkwood, 2005). Furthermore, positive allosteric modulators of mGluR5 were associated with enhanced cognitive performance in preclinical models (Balschun, Zuschratter, & Wetzel, 2006; Uslaner et al., 2009).

However, in a situation like Alzheimer's disease where synapses are lost, mechanisms of long‐term depression (LTD) may be additionally detrimental to memory function. Indeed, mGluR5 was involved in Abeta‐monomer dependent LTD (Chen et al., 2013). Most importantly, mGluR5 signaling is involved in Abetao‐dependent synaptotoxicity in preclinical models of Alzheimer's disease, and genetic deletion of mGluR5 or negative allosteric modulation was found to reduce plaque pathology and synaptic loss, and to improve cognitive performance. For a review, please see Bruno et al., 2017 and Caraci et al., 2018 (Bruno et al., 2017; Caraci et al., 2018).

So it may well be that a downregulation of mGluR5 occurs in Alzheimer's disease and this may be a compensatory process.

However, in the AD group, we only identify positive associations in a way that better cognitive performance goes along with higher mGluR5 binding. Interestingly, this also includes word recall, a function associated with hippocampal function. In our view, this would fit better with a model, in which the postsynaptic binding sites for mGluR5 are reduced, thus reflecting synaptic loss, rather than to a compensatory mechanism. Saying this, it is clear that our data are derived from just nine AD subjects and warrant replication.

### 4.3 Differences in age and depression scores do not account for the observed effects

As age and depression scores were not balanced among groups, we included these measurements as covariates. Neither age nor depression score was associated with mGluR5 binding, and group effects were dominant in hippocampus. However, in the amygdala, the depression score showed similar effect size as group speaking for a potential association with depression in this region in addition to the group effect.

A study focusing on a comparison of late‐life depression and controls did not find differences in amygdala binding(DeLorenzo et al., 2015). Also, mGluR5 binding did not change with age in most regions. A positive association between mGluR5 binding and age in the right amygdala went in the opposite direction (J. M. DuBois et al., 2016), and thus, age could clearly not contribute to the effects of reduced mGluR5 that we observe in the AD population.

A more detailed analysis on depression and age effects is given in the Supplementary material.

### 4.4 Methodological considerations and limitations

This is an exploratory study with a low sample size, which limits the generalizability of our results. Negative findings may be solely due to the low sample size. Furthermore, no additional biomarker evidence was collected at the time of recruitment. Thus, it could be that some of the healthy controls could represent preclinical Alzheimer's disease (Sperling et al., 2011) which may attenuate the differences between patients and controls.

Both groups were not age matched, and the AD group was older than the controls. The participants in the AD group also showed higher depression scores compared to the healthy controls. Both factors have been shown to be in some studies associated with mGluR5 binding (Deschwanden et al., 2011). Statistically, we introduced both variables as covariates.

Comparisons were not corrected for multiple testing, which in our eyes would have been inappropriate for an exploratory study. For discussion on this topic, please see Althouse, 2016 (Althouse, 2016). As the mGluR5 binding differences in amygdala and hippocampus were large and observed specifically in key regions involved in early AD, we consider it very unlikely that these observations are due to chance.

Furthermore, we have not reliably assessed the smoking status of the subjects. None of the AD subjects has received a clinical diagnosis for smoking; however, we cannot rule out the possibility of subtle differences in smoking habits between the groups. Reductions in mGluR5 binding in smokers and ex‐smokers are widespread throughout the cortex and not specific for the hippocampus and the amygdala (Akkus et al., 2013). We therefore consider it very unlikely that the observed results are due to differences in smoking habits.

Next, in the Alzheimer's disease group, six subjects were on cholinesterase inhibitors and 4 subjects received escitalopram and one mirtazapine. mGluR5 expression was found minimally upregulated in neurons and astrocytes of adult mice after chronic treatment with the SSRI fluoxetine, so a putative effect of antidepressant medication would point in the opposite direction of our findings (Hertz et al., 2014). However, due to the close relationship between glutamatergic and serotonergic system (Pehrson & Sanchez, 2014), we cannot entirely exclude an effect of the antidepressant medication on our results. We could not find any literature that links cholinesterase inhibitor treatment to mGluR5, but as for the antidepressant treatment, we cannot rule out an effect of cholinesterase inhibitors on mGluR5.

We have only included estimates of perfusion to estimate potential effects of synaptic density and neuronal loss. The inverse correlation of the perfusion estimate in the hippocampus with the MMSE score points to a utility of this measurement, as this relationship was observed in FDG‐PET studies as well (Alavi, Newberg, Souder, & Berlin, 1993; Rostomian et al., 2011).

We used the established bolus‐infusion paradigm for this study (Burger et al., 2010) but these methods need cerebellar gray matter as reference region which does limit the results to the assumption that no differences in binding between both groups are present in this region at this stage of disease.

Furthermore, our results are not generalizable to mechanisms in preclinical or prodromal Alzheimer's disease, as our AD Group has high variability in clinical scores and does not cover the complete spectrum of the disease.

## 5 CONCLUSION

Using ABP, we could identify a reduction in mGluR5 binding estimated by ABP in Alzheimer's dementia in the hippocampus and the amygdala, which could be due to either losses in postsynaptic binding sites or adaptive processes in Alzheimer's disease. Further studies should ideally include direct markers of synaptic density to disentangle the mechanisms behind mGluR5 binding loss.

## CONFLICT OF INTEREST

The authors report no competing interests with respect to this publication.

## AUTHORS' CONTRIBUTION

HS, ABuck, CH, RMN, and SMA conceived and designed research; ABuchmann, PGU, CH, and SMA revised the paper; HS, EG, and AFG conducted research; AJ contributed to tracer production; RM and VT coded data, VT, ABuchmann, and AFG analyzed and interpreted data; VT and AFG wrote the initial paper and had primary responsibility for final content. All authors read and approved the final manuscript.

## Supporting information

Supplementary Material

FigS1

FigS2

FigS3

## Data Availability

Research data are not shared due to privacy/ethical restrictions.
