# Supplementary material for: Reduced uptake of [11C]‐ABP688, a PET tracer for metabolic glutamate receptor 5 in hippocampus and amygdala in Alzheimer’s dementia
Source: Brain Behav. 2020 Apr 18;10(6):e01632. doi: 10.1002/brb3.1632 (PMC7303388; doi:10.1002/brb3.1632)
Supplement: Supplementary file 1 — Supplementary Material [file BRB3-10-e01632-s001.docx]

**Supplemental Material**

Additional analysis performed on the data sample with focus on the covariates age and depression score (MADRS).

The estimated marginal means of mGluR5 DVR of all 12 regions in the repeated measured model shows after correction for covariates age and MADRS only two regions with clear differences between both groups (Amygdala and Hippocampus) (see Supplemental Figure 1).


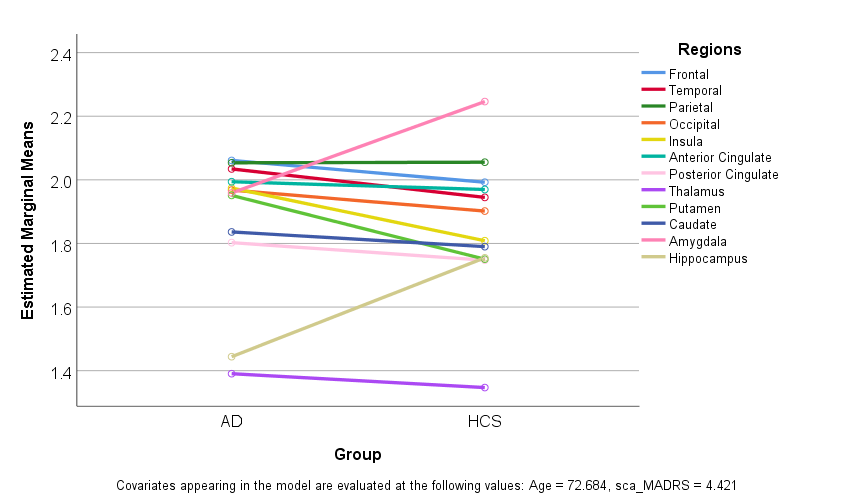


**Supplemental Figure 1**: Estimated marginal means of mGluR5 DVR as derived from the repeated measured model revealing group differences in Amygdala and Hippocampus region only.

**Additional group comparisons:**

To properly interpret the following analyses it has to be considered, that the AD patients are older and have in average higher depression scores. The maximal score in the participants was 12, which is in the range of mild depression (7-19 points (Herrmann, Black, Lawrence, Szekely, & Szalai, 1998) or 9-17 points (Muller, Szegedi, Wetzel, & Benkert, 2000)). Specifically for AD Patients a cut-off of 13 for MADRS was suggested for mild depression (Muller-Thomsen, Arlt, Mann, Mass, & Ganzer, 2005). For better interpretation supplemental Figure2 displays the distribution of age and MADRS score in both groups.


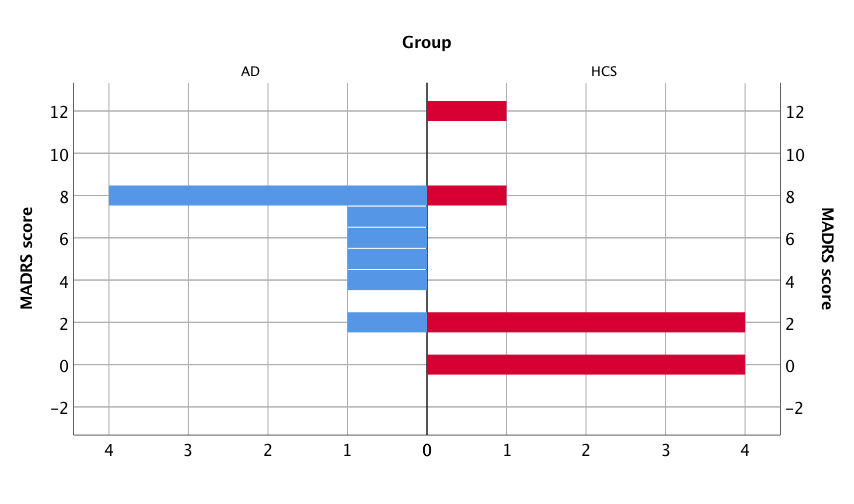


Supplemental Figure 2: Display MADRS score distribution in both groups (AD = Alzheimer’s Disease group in blue, HCS = control group in red).

We used the median split approach to generate the two age (median 75 years) and depression score groups (median score 4) in the overall sample. As both variables were higher in the AD group there were in consequence more AD Patients in the older group (6 vs. 3) and more in the higher depression score group (7 vs. 2). The results are therefore not independent from the diagnostic group effect.

**Age effect:**

Older subjects showed a significant lower mGluR5 values. This difference was significant in the Amygdala (T17=-2.138, p=0.047). It was not significant in the Hippocampus (T17=-1.717, p=0.104).

Correlating age with mGluR5 over all participants shows no significant effects (Amygdala rho=-0.365, p=0.125; Hippocampus rho=-0.454, p=0.051).

**Depression effect:**

Subjects with higher depression scores showed lower mGluR5 values, a difference which was not significant in both regions, Amygdala (T17=-2.096, p=0.051) and Hippocampus (T11.3=-1.232, p=0.243).

As the median split was below the typical cut-off for mild depression, we also tested typical cut-off. When participants were grouped according to scoring of 6 and lower and 7 and higher group no significant effect in Amygdala (T17=-1.802, p=0.089) and Hippocampus (T13.4=-1.844, p=0.087) is seen.

Correlating depression score over all participants reveals significant correlations in both regions (Amygdala rho=-0.568, p=0.011; Hippocampus rho=-0.678, p=0.001) (see Supplemental Figure 3).


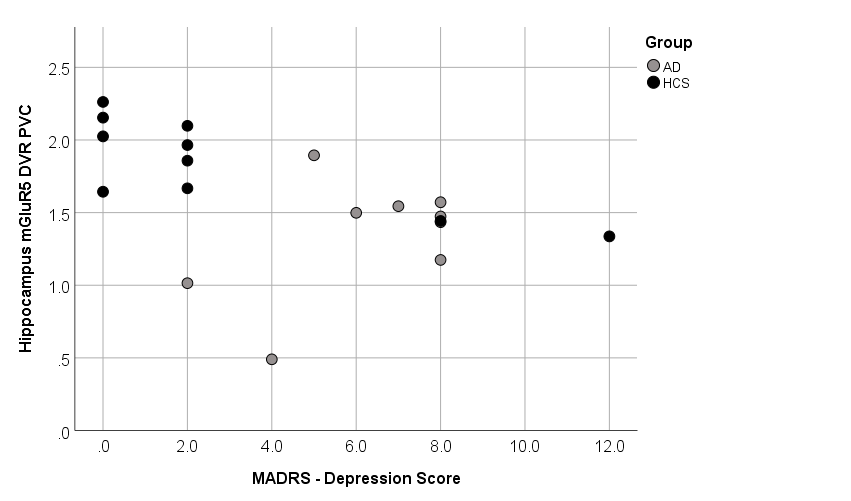


Supplemental Figure 3: Scatter plot showing significant correlation between Hippocampus mGluR5 DVR values and depression score. AD patients are displayed in grey and control group in black.

Combining above described analysis in an univariate analysis with both factors “Age group” and “MADRS group” results in no significant effect in Amygdala of neither factor MADRS group (F18,1, F= 2.038, p=0.174, PES=0.12) nor Age group (F18,1, F=2.182, p=0.160, PES=0.13), nor their interaction (F18,1, F= 0.538, p=0.474, PES=0.035). There was also no significant effect in Hippocampus factor MADRS group (F18,1, F= 0.378, p=0.548, PES=0.025), Age group (F18,1, F=1.789, p=0.201, PES=0.101), or their interaction (F18,1, F= 1.820, p=0.197, PES=0.108).

**Comparison with Literature:**

**Age effect:**

Concerning age, we have found no effect in the majority mGluR5 studies performed in Zurich except in the smoker population, where age is also confounded with smoking duration (Akkus et al., 2013). A dedicated study analyzing explicitly aging effects in healthy subjects on mGluR5 distribution (measured with ABP688) concluded that there is no age effect. This group found an effect in non PVC corrected Amygdala with higher values in the older subjects, which is neither present in PVC corrected analyses nor in direct correlation analyses (DuBois et al., 2016).

In our population presented here, we found a group differences with lower Amygdala values in older subjects, which might be due to more AD patients in the older group. The correlation over all participants did not show an age effect, which is comparable with the literature.

**Depression effect:**

Concerning depression scores and depression itself Deschwanden et al showed (Deschwanden et al., 2011) global mGluR5 binding reduction related to depression. Within the depression group a negative correlation with the depression score was shown in all regions. In a more recent study on depression also a reduction of mGluR5 (measured with 11C-ABP) binding in the MDD group compared to controls was shown (Esterlis et al., 2018). In a multimodal imaging study no mGluR5 differences as measured with 18F-FPEB PET was shown between depressive patients and controls. There was also no correlation with severity scores and mGluR5 binding in their data but a negative correlation with glutamate levels as measured with MR spectroscopy and mGluR5 DVR (Abdallah et al., 2017). In a recent study of suicidal ideation and mGluR5 binding high values were found in the post traumatic suicidal ideation group compared to the other groups including healthy control and major depression group (Davis et al., 2019). Healthy and depression groups did not show any differences in mGluR5 binding in frontal, amygdala and hippocampus region in this study using 18F-FPEB PET.

In a study on schizophrenia, the depression score did not correlate directly with mGluR5 values (Akkus et al., 2017). In non-depressed smokers, ex-smokers and nonsmokers the depression scores did not correlate with mGluR5 (Akkus et al., 2013).

Group comparisons of the depression score revealed no significant difference in our population. In line with literature, we identified lower mGluR5 values in participants with higher scores. The correlation over all subjects was significant and negative, which is also in line with literature.

**Literature:**

Abdallah, C. G., Hannestad, J., Mason, G. F., Holmes, S. E., DellaGioia, N., Sanacora, G., . . . Esterlis, I. (2017). Metabotropic Glutamate Receptor 5 and Glutamate Involvement in Major Depressive Disorder: A Multimodal Imaging Study. Biol Psychiatry Cogn Neurosci Neuroimaging, 2(5), 449-456. doi:10.1016/j.bpsc.2017.03.019

Akkus, F., Ametamey, S. M., Treyer, V., Burger, C., Johayem, A., Umbricht, D., . . . Hasler, G. (2013). Marked global reduction in mGluR5 receptor binding in smokers and ex-smokers determined by [11C]ABP688 positron emission tomography. Proc Natl Acad Sci U S A, 110(2), 737-742. doi:10.1073/pnas.1210984110

Akkus, F., Treyer, V., Ametamey, S. M., Johayem, A., Buck, A., & Hasler, G. (2017). Metabotropic glutamate receptor 5 neuroimaging in schizophrenia. Schizophr Res, 183, 95-101. doi:10.1016/j.schres.2016.11.008

Davis, M. T., Hillmer, A., Holmes, S. E., Pietrzak, R. H., DellaGioia, N., Nabulsi, N., . . . Esterlis, I. (2019). In vivo evidence for dysregulation of mGluR5 as a biomarker of suicidal ideation. Proc Natl Acad Sci U S A, 116(23), 11490-11495. doi:10.1073/pnas.1818871116

Deschwanden, A., Karolewicz, B., Feyissa, A. M., Treyer, V., Ametamey, S. M., Johayem, A., . . . Hasler, G. (2011). Reduced Metabotropic Glutamate Receptor 5 Density in Major Depression Determined by [C-11]ABP688 PET and Postmortem Study. American Journal of Psychiatry, 168(7), 727-734. doi:10.1176/appi.ajp.2011.09111607

DuBois, J. M., Rousset, O. G., Rowley, J., Porras-Betancourt, M., Reader, A. J., Labbe, A., . . . Imaging, M. (2016). Characterization of age/sex and the regional distribution of mGluR5 availability in the healthy human brain measured by high-resolution [11C]ABP688 PET. 43(1), 152-162. doi:10.1007/s00259-015-3167-6

Esterlis, I., DellaGioia, N., Pietrzak, R. H., Matuskey, D., Nabulsi, N., Abdallah, C. G., . . . DeLorenzo, C. (2018). Ketamine-induced reduction in mGluR5 availability is associated with an antidepressant response: an [(11)C]ABP688 and PET imaging study in depression. Mol Psychiatry, 23(4), 824-832. doi:10.1038/mp.2017.58

Herrmann, N., Black, S. E., Lawrence, J., Szekely, C., & Szalai, J. P. (1998). The Sunnybrook Stroke Study: a prospective study of depressive symptoms and functional outcome. Stroke, 29(3), 618-624. doi:10.1161/01.str.29.3.618

Muller-Thomsen, T., Arlt, S., Mann, U., Mass, R., & Ganzer, S. (2005). Detecting depression in Alzheimer's disease: evaluation of four different scales. Arch Clin Neuropsychol, 20(2), 271-276. doi:10.1016/j.acn.2004.03.010

Muller, M. J., Szegedi, A., Wetzel, H., & Benkert, O. (2000). Moderate and severe depression. Gradations for the Montgomery-Asberg Depression Rating Scale. J Affect Disord, 60(2), 137-140. doi:10.1016/s0165-0327(99)00162-7
