# Supplementary figures and images for: Reduced uptake of [11C]‐ABP688, a PET tracer for metabolic glutamate receptor 5 in hippocampus and amygdala in Alzheimer’s dementia
Source: Brain Behav. 2020 Apr 18;10(6):e01632. doi: 10.1002/brb3.1632 (PMC7303388; doi:10.1002/brb3.1632)

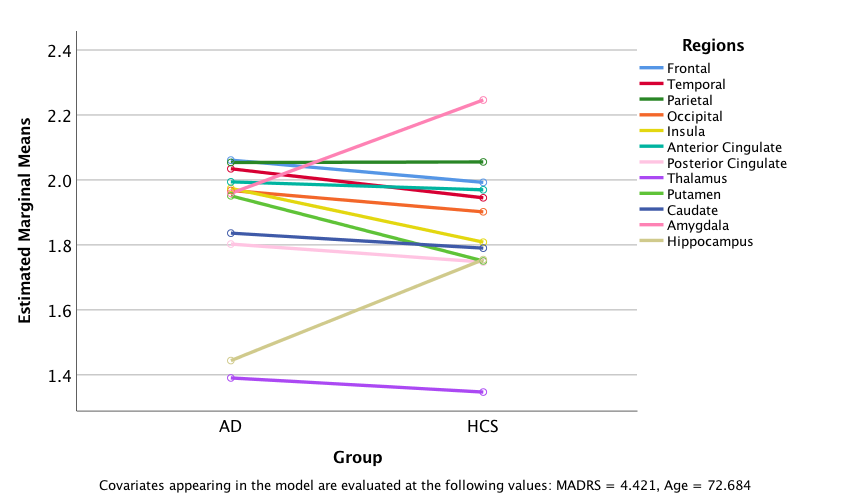

Supplement: Supplementary file 2 — FigS1 [file BRB3-10-e01632-s002.tif]

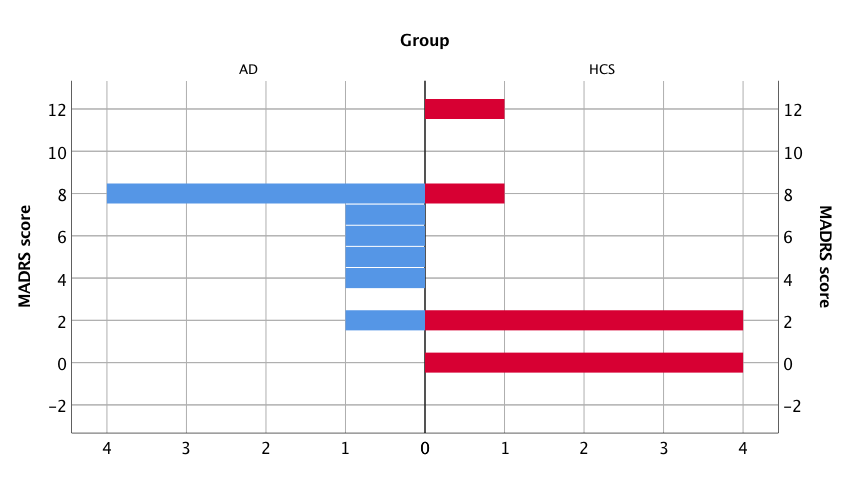

Supplement: Supplementary file 3 — FigS2 [file BRB3-10-e01632-s003.tif]

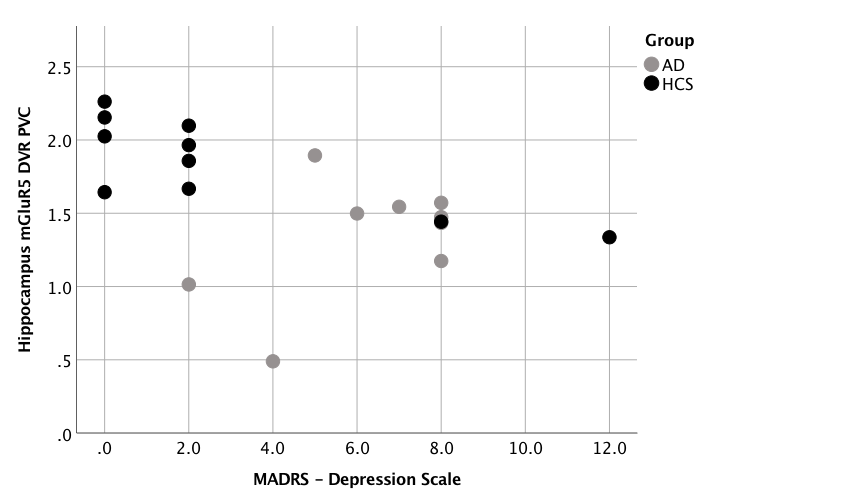

Supplement: Supplementary file 4 — FigS3 [file BRB3-10-e01632-s004.tif]
